# An *Arabidopsis thaliana* arabinogalactan-protein (AGP31) and several cationic AGP fragments catalyse the boron bridging of rhamnogalacturonan-II

**DOI:** 10.1042/BCJ20220340

**Published:** 2022-09-23

**Authors:** Dayan Sanhueza, Rifat Ara Begum, Cécile Albenne, Elisabeth Jamet, Stephen C. Fry

**Affiliations:** 1 The Edinburgh Cell Wall Group, Institute of Molecular Plant Sciences, The University of Edinburgh, Daniel Rutherford Building, The King's Buildings, Max Born Crescent, Edinburgh EH9 3BF, U.K.; 2 Laboratoire de Recherche en Sciences Végétales, Université de Toulouse, CNRS, UPS, Toulouse INP, Auzeville-Tolosane, France

**Keywords:** *Arabidopsis thaliana*, arabinogalactan-protein, borate diester bridges, pectic polysaccharide, plant cell wall, rhamnogalacturonan-II

## Abstract

Rhamnogalacturonan-II (RG-II) is a complex pectic domain in plant primary cell walls. *In vivo*, most RG-II domains are covalently dimerised via borate diester bridges, essential for correct cell-wall assembly, but the dimerisation of pure RG-II monomers by boric acid *in vitro* is extremely slow. Cationic ‘chaperones’ can promote dimerisation, probably by overcoming the mutual repulsion between neighbouring anionic RG-II molecules. Highly effective artificial chaperones include Pb^2+^ and polyhistidine, but the proposed natural chaperones remained elusive. We have now tested cationic peptide fragments of several *Arabidopsis thaliana* arabinogalactan-proteins (AGPs) as candidates. Fragments of AGP17, 18, 19 and 31 were effective, typically at ∼25 µg/ml (9–19 µM), promoting the boron bridging of 16–20 µM monomeric RG-II at pH 4.8 *in vitro*. Native AGP31 glycoprotein was also effective, and hexahistidine was moderately so. All chaperones tested interacted *reversibly* with RG-II and were not consumed during the reaction; thus they acted catalytically, and may constitute the first reported boron-acting enzyme activity, an RG-II borate diesterase. Many of the peptide chaperones became less effective catalysts at higher concentration, which we interpret as due to the formation of RG-II–peptide complexes with a net positive charge, as mutually repulsive as negatively charged pure RG-II molecules. The four unique AGPs studied here may serve an enzymic role in the living plant cell, acting on RG-II within Golgi cisternae and/or in the apoplast after secretion. In this way, RG-II and specific AGPs may contribute to cell-wall assembly and hence plant cell expansion and development.

## Introduction

**Figure 1.**
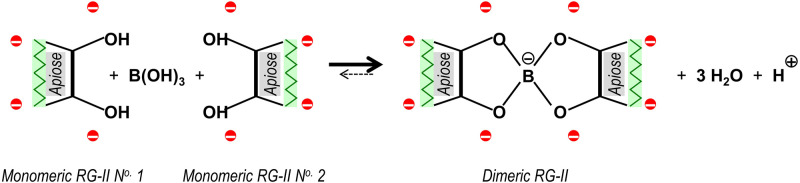
The essential reaction involved in boron bridging of RG-II. Part of the neutral apiose residue of side-chain A (in each of two monomeric RG-II molecules) is shown. The charges of its four near-neighbouring anionic sugar residues are rendered as red circles (two α-GalA residues in the RG-II backbone plus an α-GalA and a β-GalA residue attached to the rhamnose adjacent to the apiose). Monomeric RG-II has a total of ∼11 additional anionic sugar residues, giving it a net charge of ∼−14 at the pH (4.8) used in our experiments. It is evident that the close approach of two RG-II monomers for boron bridging requires the overcoming of considerable electrostatic repulsion, and dimerisation introduces an additional negative charge on the previously neutral boron atom.

Rhamnogalacturonan-II (RG-II) is a functionally important pectic domain of the plant primary cell wall. It is relatively small (∼5 kDa in its monomeric form), but has an exceedingly complex and well-conserved structure. The anionic backbone of RG-II comprises seven to ten (1 → 4)-linked α-d-galacturonic acid (GalA) residues and is covalently contiguous with the backbones of homogalacturonan and RG-I. RG-II's backbone carries six side-chains (A–F), four of which are themselves anionic [1–3]. The whole RG-II molecule is thus highly negatively charged at physiological pH in the apoplast (often pH < 5 [4]) or Golgi cisternae (often pH ≍ 6 [5]). Intriguingly, two RG-II domains can be covalently cross-linked to each other via a borate diester bond between the apiose residue of their ‘A’ side-chains [6], leading to a molecular mass of ∼10 kDa (Figure 1). The resulting dimerisation of RG-II can be demonstrated by column chromatography [7,8] or by polyacrylamide gel electrophoresis (PAGE) [9]. It is its ability to undergo boron bridging that supports the claim that RG-II is biologically important: failure to make these bridges, either because of boron deficiency or because of a mutation compromising the structure of side-chain ‘A’, results in defective cell growth and changes in cell-wall porosity [10–15].

Since RG-II is highly negatively charged, it may be difficult for pairs of RG-II molecules to approach closely enough to achieve boron bridging. Indeed, when an aqueous solution of RG-II is simply mixed with boric acid *in vitro*, almost no dimerisation occurs [16,17]). Boron bridging can, however, be very strongly enhanced in the presence of boric acid by the addition of appropriate cationic ‘chaperones’. It is assumed that these cancel the negative charges of RG-II and thus permit two RG-II molecules to come close enough for boron bridging. Highly effective chaperones (in reaction mixtures containing 500 µM monomeric RG-II and 1.2 mM boric acid, buffered at pH 3.3) are the metal cations Pb^2+^, Sr^2+^ and Ba^2+^ at 0.5 mM, whereas Ca^2+^, Cd^2+^, Cu^2+^, Mg^2+^, Ni^2+^ and Zn^2+^ are almost ineffective at this concentration) [16]. However, since these are not essential elements for plants, they cannot be the natural chaperones responsible for RG-II dimerisation *in vivo*. Ca^2+^ also works as a chaperone, but only at much higher (probably non-physiological) concentrations [16].

In attempts to discover the natural chaperone(s) responsible for dimerising RG-II *in vivo*, various other cations were tested [17]. A cationic extensin (glycoprotein rich in hydroxyproline tetraarabinoside) was effective, but the most successful chaperone found was polyhistidine (degree of polymerisation ∼100). Polyhistidine does not occur naturally; however, some arabinogalactan-proteins (AGPs) are rich in histidine and/or other cationic amino acid residues and may serve as natural chaperones, enabling the covalent reaction shown in Figure 1.

Like RG-II, AGPs have crucial biological functions in plants. For example, they appear to promote cell proliferation [18,19], cell expansion [20], xylem differentiation [21–23], somatic embryogenesis [24], pollen-tube growth [25], apoptosis [26], and cell–cell recognition [27,28]. Arabidopsis *agp19* mutants, for instance, have smaller, rounder, paler-green leaves than the wild-type, decreased stem growth, and fewer siliquae and seeds; and there were abnormalities in cell expansion, cell division and cell shape [29]. These abnormal phenotypes, especially in development, are compatible with the suggestion that some of the biological roles of AGPs and RG-II might share a common basis — for example, that certain AGPs might facilitate RG-II dimerisation.

The arabidopsis genome encodes 85 putative AGPs, some of which are highly cationic and all of which are processed (e.g. glycosylated) in the Golgi system [30]. Significantly, this is the same compartment where RG-II biosynthesis occurs [31,32] and the predominant site of boron bridging *in vivo* [33]. Furthermore, AGPs often acquire a glycosylphosphatidylinositol (GPI)-anchor, attaching them to lipid rafts of the plasma membrane [30,34,35] — another proposed site of RG-II dimerisation [36]. Thus, AGPs are at least transiently present in the right places to serve as chaperones responsible for RG-II cross-linking.

Therefore, we have now tested four cationic (His- or Lys-rich) peptide fragments of arabidopsis AGPs (AGP17, AGP18, AGP19 and AGP31; Table 1, Supplementary Figure S1) for ‘polyhistidine-like’ chaperone activity capable of dimerising RG-II *in vitro*. Other than these specific peptide fragments, AGP17, 18 and 19 are not cationic; however, much of the length of AGP31 is very rich in Lys, with many of the lysines occurring regularly every 4 or 8 residues (Table 1). There is evidence from *in-vitro* interactions on nitrocellulose membranes that AGP31 can bind to pectic components [37]. Proteins with His- and/or Lys-rich domains are not unique to arabidopsis, but also occur in most, probably all land plants. For example, proteins homologous to AGP31 can be found in flowering plants like *Gossypium hirsutum* (GhAGP31 [38]) and *Daucus carota* (DcAGP1 [39]). His/Lys domains can also be found in proteins of plants of more ancient lineages like the Equisetales (onekp|CAPN_scaffold_2004677_Equisetum_diffusum), Lycopodiales (onekp|PQTO_scaffold_2013256_Lycopodium_deuterodensum.2) and Psilotales (onekp|QVMR_scaffold_2003208_Psilotum_nudum). Interestingly, the presence of boron-bridged RG-II was described in all these plant orders [40].

**Table 1:** Cationic peptides and proteins exhibiting chaperone activity, catalysing RG-II dimerisation

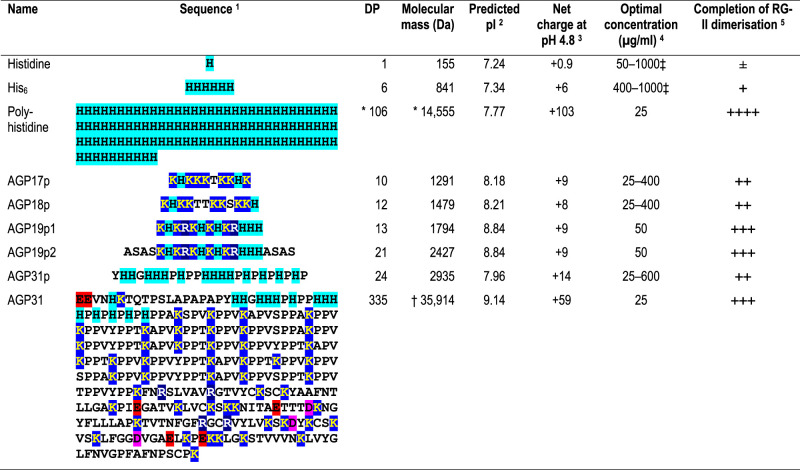

* Mean of a polydisperse preparation;

† Calculated after removal of N-terminal signal sequence but without proline hydroxylation and glycosylation. The native glycoprotein was tested for chaperone activity;

‡ No supra-optimal concentration was found;

1 Amino acid residues with cationic and anionic side-chains are highlighted in shades of blue/cyan and red/magenta respectively;

2 Isoelectric point predicted by IPC 2.0 (‘peptide’ if DP < 60, ‘protein’ if DP > 60): www.ipc2-isoelectric-point.org [47];

3 Net charge at pH 5.5 predicted by IPC 2.0 (‘peptide’ if DP < 60, ‘protein’ if DP > 60): www.ipc2-isoelectric-point.org [47];

4 Where a single value is given, this was a well-marked optimum and higher concentrations were less effective;

5 After 16–24 h at optimal concentration.

The mRNA expression of *AGP17*, *AGP18* and *AGP19* is tissue-specific, suggesting roles in growth and development: for example *AGP17* is maximally expressed in seedlings, rosette-leaves, stems and flowers; *AGP18* in roots, rosettes, stems and flowers; and *AGP19* in roots, stems and flowers [29]. AGP31 is an abundant glycoprotein in cell walls of etiolated hypocotyls [41].

In this work, we tested the chaperone activity of the His- and/or Lys-rich peptides of the four mentioned AGPs as well as the native glycoprotein AGP31 and some related molecules (Table 1), and report that they do indeed promote the boron bridging of RG-II in the presence of boric acid *in vitro*. We discuss the possibility that these molecules could actually be the first enzymes involving boron compounds to be described.

## Results

### Histidine, hexahistidine and polyhistidine as potential chaperones in RG-II dimerisation

**Figure 2.**
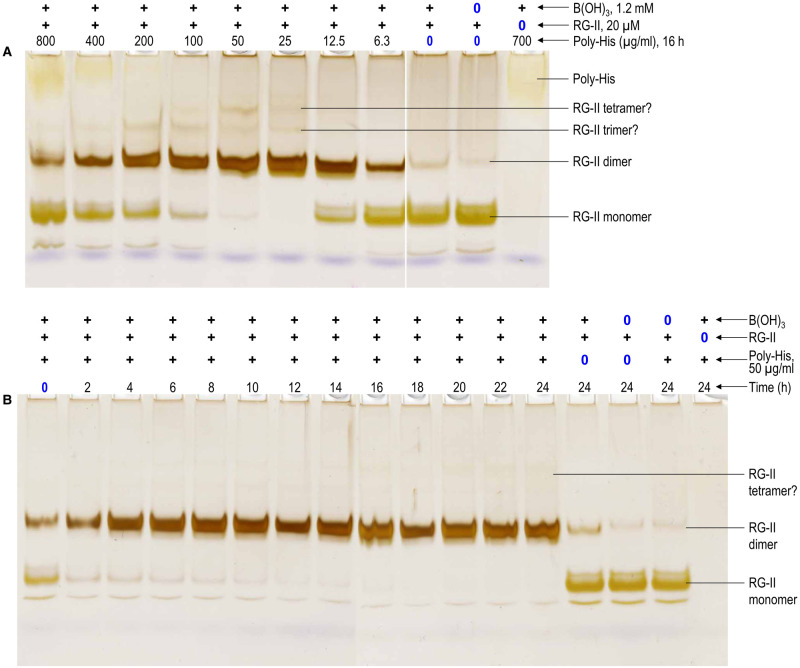
Polyhistidine as a cationic chaperone, catalysing the boron bridging of RG-II. (**a**) The reaction mixture contained 100 µg/ml RG-II monomer (≍20 µM), 1.2 mM boric acid, 0–800 µg/ml (≍0–44 µM) polyhistidine.Cl^−^, and 50 mM acetate (Na^+^) buffer pH 4.8. Controls lacked boric acid or RG-II, as indicated above the gels. After 16 h, 0.8 µg of the RG-II was analysed by PAGE followed by silver staining. (**b**) As (**a**) but constant 50 µg/ml polyhistidine and varying incubation time (0–24 h). The bands are quantified in Figure 3.

**Figure 3.**
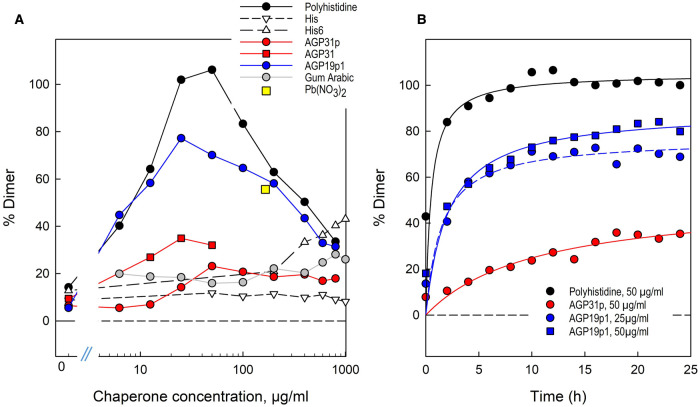
Quantification of chaperone-dependent RG-II dimerisation. Silver-stained gels (shown in Figures 2, 4–7 and Supplementary Figure S6) were scanned and the luminosity of the monomer and dimer bands was estimated in Photoshop. The proportion of RG-II dimerised was thereby estimated (see Supplementary Figures S2, S3). In (**a**), the incubation time was 16 h [the single 0.5 mM lead nitrate sample is plotted at 166 µg/ml on the *x*-axis]. In (**b**), the curves are rectangular hyperbolae, fitted to the datapoints by SigmaPlot.

It was known that polyhistidine (DP ∼ 10^2^) at 100–700 µg/ml favoured the dimerisation of monomeric RG-II in the presence of boric acid [17]. In the present work, the dose–response relationship was investigated in greater detail (Figures 2a, 3a). In the absence of polyhistidine, boric acid caused little dimerisation. We consistently found that the optimal polyhistidine concentration was ∼25–50 µg/ml (1.4–2.8 µM), which brought about the essentially complete dimerisation of a 7–14-fold molar excess of RG-II within 16 h of incubation. Higher concentrations (>200 µg/ml) gave much weaker dimerisation. Polyhistidine was effective only when the concentration of boric acid exceeded that of RG-II (∼20 µM; Supplementary Figure S4).

Polyhistidine has a predicted pI of ∼7.8 (Table 1), so at the pH of the gel buffer (8.8) it had a slight net negative charge (entirely due to the C-terminal −COOH group). This, together with its relatively large size (14.6 kDa), caused polyhistidine to migrate only a short distance into the gel. It stained weakly with Ag^+^ (Figure 2a).

Interestingly, moderate concentrations of polyhistidine (25–200 µg/ml) provoked the formation of faint, slower-moving RG-II bands that might be interpreted as trimers and tetramers of RG-II (Figure 2a; also detectable in Supplementary Figure S5). These bands are unlikely to be RG-II–polyhistidine complexes because they became less abundant at the highest polyhistidine concentrations. We tried to elute the putative trimers and tetramers but they appear to change into dimers during the elution process. Nevertheless, it is difficult to envisage them being other than RG-II-derived, given the ingredients used in our reaction mixtures.

Time-course studies revealed that the dimerisation of RG-II caused by 50 µg/ml polyhistidine was initially very rapid, almost reaching completion in the first 2–4 h; thereafter, dimerisation was considerably slower, but did go to completion within ∼16 h (Figures 2b, 3b).

**Figure 4.**
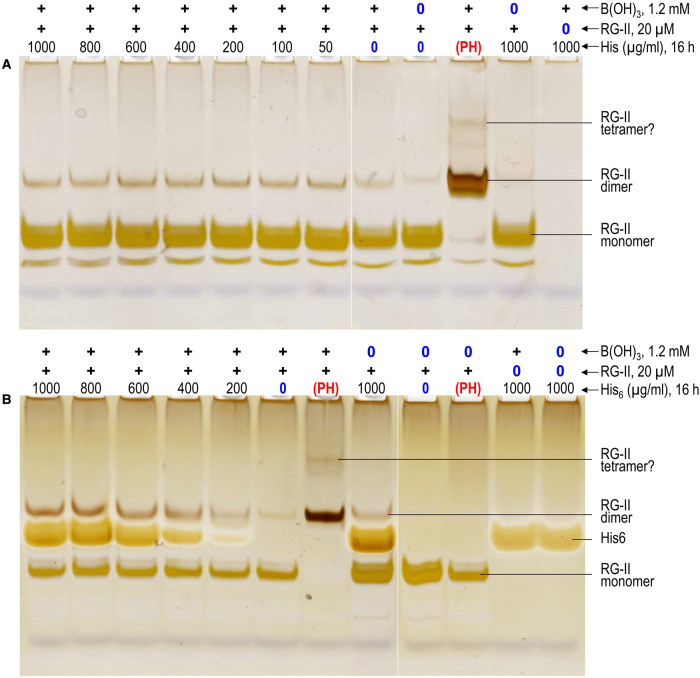
Concentration dependence of histidine and hexahistidine as potential chaperones. The reaction mixtures contained 100 µg/ml RG-II monomer (≍20 µM), 1.2 mM boric acid, 0–1000 µg/ml histidine (0–6450 µM) (**a**) or His_6_.Cl^−^ (His_6_; 0–966 µM) (**b**), and 50 mM acetate (Na^+^) buffer pH 4.8. Controls lacked boric acid or RG-II, as indicated above the gels. Other controls contained 50 µg/ml polyhistidine.Cl^−^ (PH) instead of His or His_6_. After 16 h at 20°C, 0.8 µg of the RG-II was analysed by PAGE followed by silver staining. The bands are quantified in Figure 3.

Although polyhistidine was an excellent chaperone able to boron-bridge RG-II, monomeric histidine (0.16 kDa; pI = 7.2) had only very slight chaperone activity compared with the histidine-free control. Any effect of monomeric histidine was approximately equal at all concentrations tested (50–1000 µg/ml; Figures 3a, 4a).

His_6_, which is intermediate between histidine and polyhistidine, showed some chaperone activity, moderately promoting RG-II dimerisation, especially at concentrations of 400–1000 µg/ml; no supra-optimal concentration was found (Figures 3a, 4b). In this experiment, as in some others, a small amount of dimerisation occurred in the absence of chaperones, and even in the absence of deliberately added boron; however, this slight background activity does not invalidate the above main conclusions. Unlike histidine, His_6_ was stainable on polyacrylamide gels, migrating between RG-II monomer and dimer though not modifying their mobilities. Thus the RG-II–His_6_ complex (as with all RG-II–chaperone complexes tested in this work) was very short-lived compared with the electrophoresis run-time. The observed electrophoretic mobility towards the anode agrees with the prediction that His_6_ (pI ≍ 7.3) has a net negative charge during PAGE (gel buffer pH 8.8).

### AGP31 and its histidine-rich peptide as chaperones promoting RG-II

**Figure 5.**
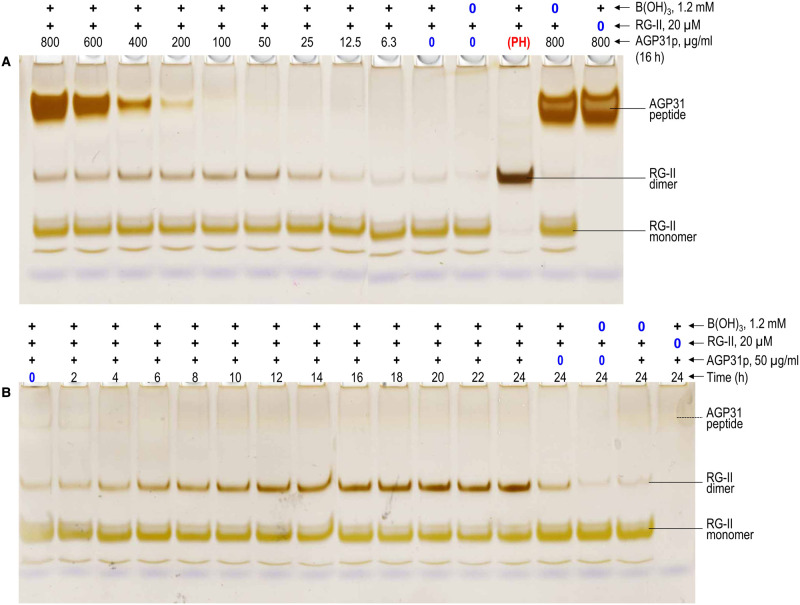
AGP31p as a cationic chaperone, catalysing the boron bridging of RG-II. (**a**) The reaction mixtures routinely contained 100 µg/ml RG-II monomer (∼20 µM), 1.2 mM boric acid, 0–800 µg/ml (0–273 µM) AGP31p, and 50 mM acetate (Na^+^) buffer pH 4.8. Controls lacked boric acid or RG-II, as indicated above the gels. The polyhistidine.Cl^−^ (PH) for the sample in lane 12 was at 50 µg/ml. After 16 h, 0.8 µg of the RG-II was analysed by PAGE followed by silver staining. (**b**) As (**a**) but constant 50 µg/ml AGP31p and varying incubation time (0–24 h). The bands are quantified in Figure 3.

Although polyhistidine is an excellent chaperone, causing the boron bridging of RG-II, it is not a naturally occurring protein. However, plants do possess certain highly cationic (glyco)proteins, especially AGPs, that have sequences very rich in histidine. For example, arabidopsis AGP31 includes the sequence YHHGHHHPHPPHHHHPHPHPHPHP near its *N*-terminus (Supplementary Figure S1; Table 1; referred to here as AGP31p), which we synthesised and tested for chaperone activity. It was moderately effective at promoting RG-II dimerisation within 16 h (Figures 3b, 5b). The strongest effect was observed at ∼50 µg/ml; higher concentrations up to 800 µg/ml had very similar effects (Figures 3a, 5a). RG-II dimerisation by AGP31p was gradual over a 24 h period but never approached completion; over the first ∼12 h, dimerisation was approximately linear but reached only ∼30% completion within 12 h (Figures 3b, 5b). In a similar experiment, 50 µg/ml polyhistidine was more effective and faster acting (almost 100% dimerisation within 4 h; Figures 2b, 3a) than AGP31p.

**Figure 6.**
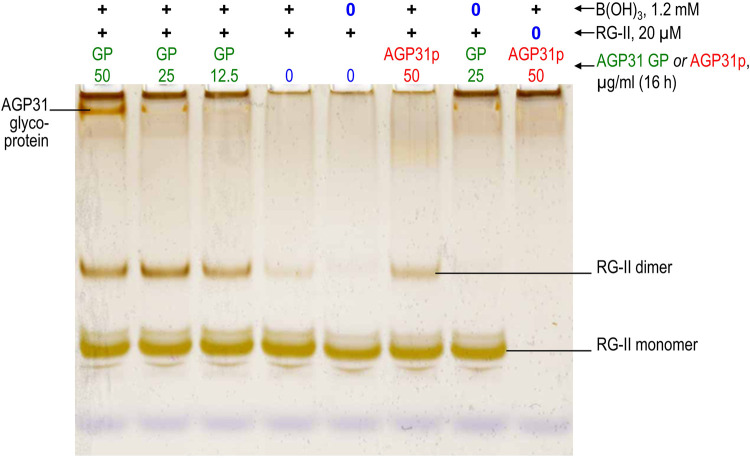
Native AGP31 glycoprotein compared with its peptide fragment AGP31p, as cationic chaperones, catalysing the boron bridging of RG-II. Reaction mixtures contained 100 µg/ml RG-II monomer (≍20 µM), 1.2 mM boric acid, 0–50 µg/ml (i.e. up to roughly 0.7 µM) AGP31 glycoprotein (GP) or 50 µg/ml (17 µM) AGP31p, and 50 mM acetate (Na^+^) buffer pH 4.8. Controls lacked boric acid or RG-II. After 16 h at 20°C, 0.6 µg of the RG-II was analysed by PAGE followed by silver staining. The loading in the left-hand lane contained 0.3 µg AGP31 glycoprotein. The bands are quantified in Figure 3.

Native AGP31 glycoprotein (∼50–90 kDa [41]) also exhibited chaperone activity, with an optimal concentration of ∼25 µg/ml (roughly 0.2–0.5 µM; Figures 6, 3a). The molar concentration is difficult to state with precision because of glyco-heterogeneity. However, the native glycoprotein is clearly effective at dimerising RG-II at a much lower molar concentration than the peptide, AGP31p (∼17 µM; Figure 5).

### Cationic peptide fragments of AGP19 are also powerful RG-II chaperones

**Figure 7.**
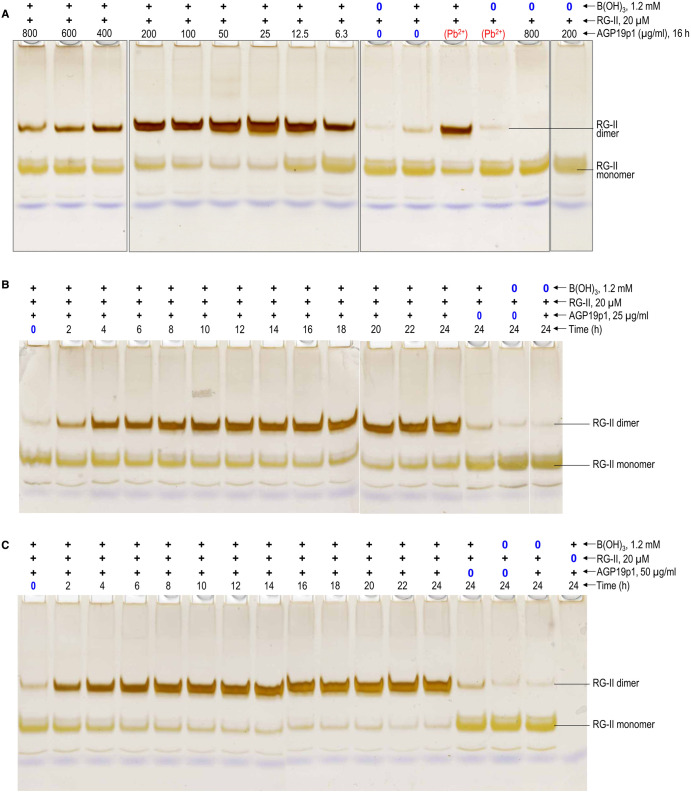
AGP19p1 action as an RG-II chaperone, catalysing the boron bridging of RG-II. Reaction mixtures contained 100 µg/ml RG-II monomer (≍20 µM), 1.2 mM boric acid, 0–800 µg/ml (0–446 µM) AGP19p1 (tridecapeptide), and 50 mM acetate (Na^+^) buffer, pH 4.8 at 20°C. (**a**) Various AGP19p1 concentrations [0–800 µg/ml (0–446 µM)]; always 16 h incubation. (**b**) 50 µg/ml (28 µM) AGP19p1, 0–24 h incubation; (**c**) 25 µg/ml (14 µM) AGP19p1, 0–24 h incubation. Controls lacked boric acid, RG-II or AGP19p1, or contained 0.5 mM Pb^2+^ as an alternative chaperone. After incubation, 0.8 µg of the RG-II was analysed by PAGE followed by silver staining. The bands are quantified in Figure 3.

Another cationic AGP of arabidopsis is AGP19, whose sequence (Supplementary Figure S1) contrasts strongly with that of AGP31. An extremely cationic tridecapeptide fragment of AGP19 (AGP19p1; KHKRKHKHKRHHH; Table 1) was found to be an excellent chaperone, the optimal concentration for RG-II dimerisation being 25–50 µg/ml, leading to ∼75% dimerisation (14–28 µM; Figures 3a, 7a). Higher concentrations were less effective, as noted for polyhistidine. As revealed by the dimer:monomer ratios visible in Figure 7a and Figure 3a, AGP19p1 was somewhat more effective than 0.5 mM Pb(NO_3_)_2_, which has been widely used as a [non-biological] model chaperone [42]. AGP19p1 was not detected by silver staining of gels because with a pI of 8.8 it would not be expected to migrate into the gel.

Although an effective chaperone at low concentrations, AGP19p1 did not take the dimerisation of RG-II to completion (Figures 3, 7), unlike polyhistidine. The dimerisation was rapid in first 2 h; thereafter, the process continued slowly (most easily seen by the gradual decrease in RG-II monomer) but never reached completion (Figures 3b, 7b,c).

**Figure 8.**
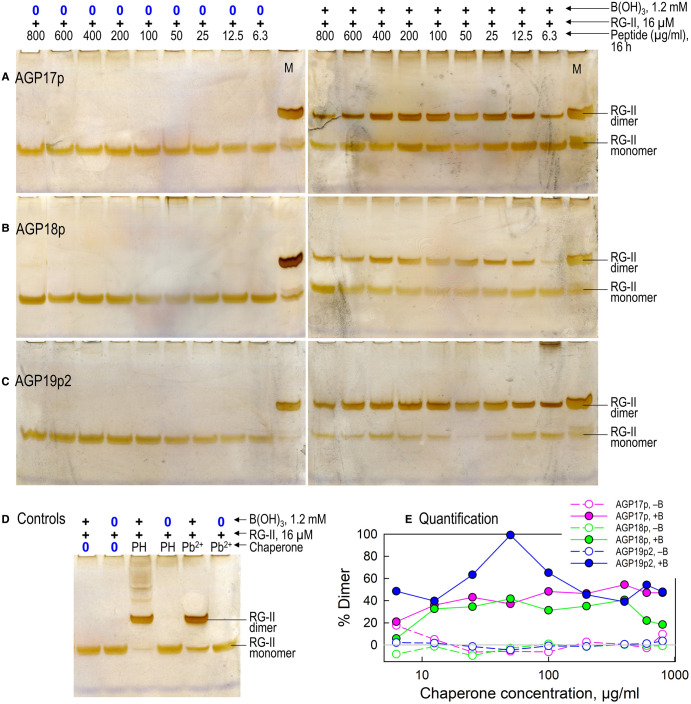
AGP17p, AGP18p and AGP19p2 as chaperones, catalysing the boron bridging of RG-II. Reaction mixtures contained 80 µg/ml RG-II monomer (≍16 µM), 0 or 1.2 mM boric acid, 6.3–800 µg/ml* of an AGP peptide, and 50 mM acetate (Na^+^) buffer, pH 4.8. Left-hand gels: controls lacking boric acid. Peptides were: a, AGP17p; b, AGP18p; c, AGP19p2. Panel (d) shows incubations conducted with 0.5 mM Pb^2+^, 50 µg/ml polyhistidine (PH) or no chaperone instead of the AGP peptides. After 16 h at 20°C, 0.8 µg of the RG-II was analysed by PAGE followed by silver staining. Marker lanes (M) contained 1 µg monomeric RG-II + 1 µg dimeric RG-II. Panel (e) shows a quantification of the proportion of RG-II dimerised in the presence and absence of boric acid (filled and empty symbols respectively), as estimated from scans of the stained gels shown in (**a**)–(**c**). *The highest (800 µg/ml) peptide concentrations represent ∼620 µM AGP17p, 540 µM AGP18p, and 320 µM AGP19p2.

The above peptide, AGP19p1, differs from native AGP19 in, among other ways, lacking the neutral, glycosylated hydrophilic residues which normally flank the highly cationic KHKRKHKHKRHHH sequence. We therefore produced another AGP19 peptide (ASASTKHKRKHKHKRHHHASAS; AGP19p2; Table 1), whose two neutral ASAS flanks were designed to mimic the neighbouring hydrophilic hydroxyproline-containing sequences present in the full sequence of AGP19 (Supplementary Figure S1). The modified peptide, AGP19p2, also functioned as a chaperone, bringing about RG-II dimerisation, again with a distinct optimum concentration (∼50 µg/ml; Figure 8c,e), but with little if any improvement over the ‘naked’ cationic sequence, AGP19p1. A direct comparison of AGP19p1 versus AGP19p2 is shown in Supplementary Figure S5.

### Cationic peptides of AGP17, AGP18 and are also RG-II chaperones

Arabidopsis AGP17 and AGP18 (related to AGP19) also include highly cationic peptide stretches (Table 1). We found that 50 µg/ml AGP17p and AGP18p mimic AGP19p2 as chaperones, dimerising RG-II (Figure 8); the results are compared with those with Pb^2+^ and polyhistidine in Supplementary Figure S5.

AGP17p and AGP18p were effective over a broad range of concentrations, 12–400 µg/ml, but were less effective at the lowest and highest concentrations tested (6 and 800 µg/ml; Figure 8a,b,e). However, neither of them was as effective as AGP19p2 — most easily demonstrated by its ability (at 50 µg/ml) to effect the almost complete disappearance of RG-II monomer (Figure 8c,e). No concentration of AGP17p and AGP18p took RG-II dimerisation to completion within 16 h.

The controls (Supplementary Figure S5) show that the monomeric RG-II preparation lacked contaminating dimer. Polyhistidine was again able to dimerise RG-II completely and Pb^2+^ promoted it very well albeit not to completion. AGP peptides in the absence of boric acid did not form dimers.

### Gum arabic AGP and its core protein have little chaperone activity

So far we have focused on AGPs with cationic stretches and observed good chaperone activity. We next tested a non-cationic AGP to discover whether the chaperone activity was due to the intrinsic nature of AGP or to the net positive charge. A much-studied and commercially available AGP is gum arabic. This is a glycoprotein composed of ∼2% protein and ∼98% carbohydrate. The protein backbone has roughly equal proportions of acidic and basic amino acid residues, and the carbohydrate chains contain ∼20% glucuronic acid plus 4-*O*-methylglucuronic acid residues [43]; thus the overall glycoprotein is highly acidic. The native gum arabic glycoprotein had slight chaperone activity (Supplementary Figure S6a; Figure 3a) but fragments of the protein core, released by acid hydrolysis of the sugar residues, had almost no chaperone activity (Supplementary Figure S6b). The ‘1000*' loadings (right-hand lanes), contained gum arabic (or its protein core) plus polyhistidine, and show that these components did not produce bands that would interfere in the detection of RG-II monomer and dimer. However, it was not possible to quantify the dimeric bands in Supplementary Figure S6b owing to the presence of neighbouring gum arabic protein fragments.

## Discussion

### Size matters: large histidine peptides outperform small ones as chaperones

RG-II undergoes very little dimerisation when mixed with boric acid *in vitro* in the absence of chaperones, but several substances tested here were able to catalyse boron bridging. We confirmed the ability of polyhistidine and Pb^2+^ to act as cationic chaperones, assumed to allow polyanionic RG-II molecules to overcome their mutual electrostatic repulsion and approach closely enough for bridging via a borate diester bond.

The polyhistidine used (mean DP 106) was a highly effective chaperone, catalysing the complete dimerisation of RG-II (Figures 2a, 3a). At the other extreme, monomeric histidine did not function as a chaperone (Figure 4a). To explain this, consider that each RG-II molecule has a net charge of roughly −14 at pH 4.8, as used in our reaction mixtures. Presumably the RG-II–histidine ionic bonds were too short-lived to simultaneously cancel enough of the negative charges on the RG-II monomer (20 µM) to enable dimerisation, even in the presence of up to a 320-fold excess (6400 µM) of histidine. Indeed, RG-II–histidine ionic bonds may be compared with the RG-II–Na^+^ ionic bonds which would have been present in all reaction mixtures owing to the 1250-fold molar excess (25 mM) of Na^+^ from the acetate buffer. Thus small ions such as histidine and Na^+^ do not work. Between polyhistidine and histidine, His_6_ was a moderately effective chaperone at His_6_:RG-II molar ratios between 20 : 1 and 50 : 1 (the highest ratio tested; Figures 3a, 4b).

### Towards the discovery of natural chaperones

Although polyhistidine is an excellent artificial chaperone, this polypeptide does not occur naturally. As a step towards the discovery of natural chaperones, we therefore tested several His- and/or Lys-rich peptide sequences known from arabidopsis AGPs which are expected to be present in the apoplast and Golgi system — the feasible sites of RG-II dimerisation *in vivo*. We have shown that several such cationic peptides indeed possess chaperone activity, catalysing RG-II dimerisation: AGP17p, AGP18p, AGP19p1, AGP19p2 and AGP31p, whose sequence, size, isoelectric point and observed chaperone activity are listed in Table 1. Gum arabic (an AGP lacking a cationic stretch) and its peptide core exhibited little or no RG-II chaperone activity (Supplementary Figure S6). We conclude that the peptides of AGP17, 18, 19 and 31 owe their chaperone activity to their cationic nature rather than solely to any intrinsic activities as AGPs — for example an ability to bind ‘Yariv antigens’ (certain tri-β-glycosylated phenylazo compounds [45]).

AGP31p, a fragment of the glycoprotein AGP31 (which is abundant in rapidly growing etiolated hypocotyl cell walls), acted slowly as a chaperone (Figure 3b). It did not dimerise RG-II to completion and was thus not as effective as polyhistidine. However, AGP31 itself (the native glycoprotein) also acted as a chaperone, catalysing RG-II dimerisation to somewhat nearer completion than did the peptide at same (w/v) concentrations (12.5–50 µg/ml; Figuress 3a, 5a, 6). The optimal concentration of the native glycoprotein was 0.2–0.5 µM (25 µg/ml; Figure 3a), much lower than the tested molar concentrations of the peptides. Thus, the naturally occurring AGP31 glycoprotein is relatively effective as an RG-II chaperone even at very low molar concentrations (∼0.2–0.5 µM) which might be biologically relevant.

Native AGP31 is highly cationic (Table 1; Supplementary Figure S1). The molecular mass of the glycoprotein is higher than the quoted value of 35.9 kDa (Table 1) because many of the proline residues are hydroxylated and *O*-glycosylated [41], and at least one Asn residue is *N*-glycosylated [44]. Indeed, AGP31 exists *in vivo* as a broad range of sizes (∼50–90 kDa) [41]. Full-length AGP31 possesses numerous basic residues (50 Lys, 4 Arg, 15 His), whereas the acidic residues are 3 Asp, 5 Glu and at most 2 GlcA [41], supporting its description as a polycation. AGP31, synthesised in the endomembrane system, is ultimately targeted to the apoplast. Thus it exists in the right places to make a significant contribution to the boron bridging of RG-II *in vivo*. It is particularly relevant that AGP31 can physically interact *in vitro* with the pectic domains RG-I and homogalacturonan [37], though this has not yet been directly demonstrated for RG-II.

Other arabidopsis AGPs possessing cationic stretches are AGP17, AGP18 and AGP19. We found that fragments of these (AGP17p, AGP18p, AGP19p1 and AGP19p2) were effective chaperones catalysing RG-II dimerisation, typically at concentrations of 25–400 µg/ml (Figs. 7–8; Table 1).

The gene encoding the backbone of AGP19 is expressed (at the level of mRNA accumulation) in arabidopsis cell-suspension cultures and in all organs examined *in planta*, the order of mRNA abundance being stems > flowers > roots > leaves [29]. Its promoter drove GUS expression particularly in the vasculature of leaves, roots, stems, flowers, styles and siliques. An *agp19* null mutant had small, round, flat rosette-leaves, delayed growth, and short final lengths of hypocotyls and stems. On the abaxial leaf surface, the mutant had epidermal cells on average 0.73× the area of those of the wild-type; conversely, the mutant had 1.25× more epidermal cells per mm^2^ of leaf [29], indicating that the mutation predominantly interferes in cell expansion. Likewise in the palisade and spongy mesophyll layers of leaves, the mutant had smaller cells (as seen in paradermal sections) and bigger air spaces. The expression profiles of AGP mRNAs and the phenotypes of *agp* mutants suggest key roles in cell expansion [20,29], which are compatible with AGPs’ proposed roles in cell-wall biochemistry, such as catalysing the boron bridging of RG-II.

AGP17, AGP18 and AGP19 are each predicted to have a GPI anchor that will attach the glycoprotein to the outer face of the plasma membrane. This positioning is relevant because most RG-II dimerisation seems to occur either (a) very soon after secretion [9,36], thus probably in the vicinity of the plasma membrane, or (b) within the Golgi endo-membrane system [33], where AGPs are glycosylated. Therefore, at the likely sites of RG-II dimerisation, cationic AGP domains would be available to participate as chaperones catalysing boron bridging.

AGPs 17, 18 and 19 each possess one highly cationic domain: AGP17p, AGP18p and AGP19p respectively (Supplementary Figure S1, Table 1). Excluding these remarkable cationic domains, the glycoproteins differ from each other ionically — the basic:acidic amino acid ratios (after removal of the signal sequence) being 5 : 13, 4 : 10 and 1 : 0 in AGP17, AGP18 and AGP19 respectively. This difference may enable the three glycoproteins to serve different biologically relevant roles that involve ionic interactions, e.g. with RG-II or other pectic domains. The glycosylation of AGP19 does not appear to have been studied in detail, but extracted AGP19 has a wide range of molecular mass, ∼75–200 kDa (Figure 7 of Yang et al. [46]), indicating that it bears a very high proportion (∼80–90%) of carbohydrate. AGP17 is a glycoprotein of ∼80–150 kDa, ∼86% of the mass being carbohydrate (mainly Ara and Gal residues, with smaller proportions of Rha and GlcA [47]). The GlcA residues will decrease the pI of the whole glycoprotein and may thus also influence various biological functions *in vivo*. In the light of the observations on native AGP31 versus its peptide, AGP31p, it is possible that native AGP19 would be a better chaperone than the peptides (AGP19p1 and AGP19p2) tested so far. The same is also likely to apply to native AGP17 and 18.

The AGP and AGP peptides did not cause complete dimerisation of RG-II in our experiments (although AGP19p1 and AGP19p2 came close). Nevertheless, it is possible that they would be more effective under *in-vivo* conditions. For example, Ca^2+^ in the Golgi system or cell walls might serve as a cofactor, potentially synergising with AGPs. Also, we arbitrarily chose pH 4.8 for dimerisation experiments, whereas the natural pH at the specific site of boron bridge formation might differ from this. In addition, we used purified RG-II as substrate, whereas RG-II does not normally occur in free form *in vivo*; on the contrary, it is glycosidically linked to homogalacturonan and/or RG-I, which might help to manoeuvre the RG-II domains into an orientation favouring boron bridging. Finally, the natural boron ‘donor substrate’ may not be free boric acid, as used here, but rather an organic complex such as one with a glycosylinositol phosphorylceramide [36].

### Chaperones interact *reversibly* with RG-II to facilitate boron bridging

The chaperones tested here did not cause any apparent loss of RG-II during PAGE. In contrast, polylysine, which was tested previously [17], did cause RG-II to ‘disappear’ during PAGE by forming a long-lived RG-II–polylysine complex that migrated towards the cathode (out of the gel). Furthermore, after incubation of RG-II with any of the chaperones tested here, both the dimeric RG-II and the remaining monomer had the same electrophoretic mobility as in the absence of chaperones, indicating that the RG-II–peptide ionic complex (proposed to be the species participating in boron bridge formation) was ephemeral, and dissociated during electrophoresis. Any irreversible RG-II–chaperone complex would be expected to differ from free RG-II in mobility during PAGE since all the peptide chaperones tested are at least 25% the molecular mass of an RG-II monomer. Thus all RG-II–chaperone interactions studied here are readily reversible; the complexes dissociated during PAGE and would presumably also do so *in vivo*. This implies that the chaperones are not consumed when promoting RG-II dimerisation: they acted catalytically.

### AGPs and their peptides as boron-acting enzymes

The above considerations support the notion that AGP31 and the cationic peptides tested here can serve as boron-acting enzymes. Crucially, AGP31 is a naturally occurring protein that we have now shown is capable of catalysing a chemical reaction (Figure 1) involving a change in covalent structure between substrates and products. The AGP and peptides were not used up during the reaction, as can be seen in those cases where the chaperone is detectable on silver-stained gels (AGP31, AGP31p, polyhistidine and His_6_). The chaperones evidently bind the RG-II substrate reversibly and are released intact after catalysing the reaction. As expected for an enzyme, the larger chaperones (AGP31 and polyhistidine) can catalyse the boron-bridging reaction of an excess of RG-II: for example, 0.2–0.7 µM AGP31 functioned on 20 µM RG-II. In the case of the model chaperone, 1.4 µM polyhistidine was able to catalyse the complete dimerisation of a 14-fold molar excess of RG-II — behaviour typical of an enzyme. Furthermore, AGP19p2 (a fragment which may be a good model of an enzymic AGP) can also catalyse the almost complete dimerisation of RG-II (Figure 8e).

The reaction under investigation (Figure 1) occurs to a slight extent in the absence of a catalyst (e.g. Figures 3, 4); however, this property applies more extremely to many other well-accepted enzymic reactions such as those catalysed by carbonic anhydrase and superoxide dismutase. Moreover, the catalytic effect of AGP31 etc. on RG-II plus boric acid can be mimicked by simple inorganic agents such as Pb(NO_3_)_2_; but again the same can be said of the activities of many other enzymes e.g. carboxylesterases (mimicked by NaOH) and catalase (mimicked by MnO_2_).

The reaction (Figure 1) catalysed by AGP31 and the peptides investigated here is of the type acid + alcohol → ester + H_2_O. In the case of boric acid + RG-II, the reaction is essentially irreversible in the direction written here, unlike the corresponding reaction with most carboxylesterases, which is essentially irreversible in the opposite direction (ester + H_2_O → acid + alcohol). However, enzymes do not dictate the direction of a reaction, so we suggest that this consideration should not affect the nomenclature of the enzyme activity proposed here: it is an esterase, more specifically an RG-II borate diesterase. It is of interest that the Enzyme Commission does not currently list any enzymes acting on boron compounds (https://iubmb.qmul.ac.uk/enzyme/EC3/).

### Many peptide chaperones lose effectiveness at high concentration

Many of the chaperones tested, e.g. polyhistidine, exhibited an optimum concentration favouring boron bridging; higher and lower concentrations were less effective. At one extreme, boric acid plus RG-II in the complete absence of chaperones led to negligible dimerisation (e.g. Figure 2a), presumably because the negatively charged RG-II molecules (net charge approximately −14 at pH 4.8) mutually repelled. Conversely, in the presence of a high concentration of polyhistidine, two factors might impede dimerisation: (i) each RG-II molecule would become ionically bonded to an excess of the cationic peptide, forming a complex with a net positive charge, and such complexes would also mutually repel electrostatically, inhibiting dimerisation; (ii) only rarely would two RG-II molecules become simultaneously attached to the same polyhistidine molecule. However, at intermediate polyhistidine concentrations, there was an optimum where the complexes exhibited little electrostatic repulsion and two RG-II molecules would frequently become bonded side-by-side to the same polyhistidine molecule, enabling optimal boron bridging (Figure 2a).

His_6_ has a net charge of ∼+5 at pH 4.8 so it is expected to bond ionically to RG-II under the incubation conditions used. We suggest that the RG-II–His_6_ ionic linkage is much shorter-lived than the RG-II–polyhistidine linkage, and therefore at any moment the great majority of the RG-II molecules were not attached to enough His_6_ molecules to acquire a net positive charge whose cationic repulsion would inhibit dimerisation. This would explain why even a very high His_6_ concentration did not inhibit RG-II dimerisation.

Most of the AGP-based cationic peptides tested (AGP17p, AGP18p, AGP19p1, AGP19p2, and possibly AGP31p and the native AGP31 glycoprotein) also exhibited a similar phenomenon to that reported for polyhistidine, each exhibiting optimal concentrations (though sometimes a fairly broad range) under the experimental conditions used (Figs. 5–8; collated in Table 1 and Figure 3). This is likewise interpreted as evidence for an optimum, where the RG-II–chaperone complex has little or no net charge (and thus minimal electrostatic repulsion), and two RG-II molecules can simultaneously bind to the same glycoprotein or peptide molecule, as suggested above for polyhistidine.

### Why does chaperone-mediated dimerisation of RG-II decelerate during incubation *in vitro*?

The rate of RG-II dimerisation at optimal chaperone concentrations was often initially rapid, but later slowed down — in some cases eventually reaching completion, and in other cases not (Figure 3b). For example, the optimal polyhistidine concentration catalysed very rapid boron bridging during the first ∼2 h; thereafter the process slowed down but nevertheless reached completion. In other cases the deceleration was more extreme, and dimerisation never reached completion. For example, the boron bridging of RG-II by 50 µg/mg AGP19p1 was gradual: by 24 h, the system was approaching ∼80% complete dimerisation. At 25 µg/ml AGP19p1, ∼70% dimerisation had been achieved by 24 h and no further dimerisation appeared still to be occurring at that time.

It is unclear why the dimerisation sometimes stops before completion. We suspected that the reaction might be reaching an equilibrium, after which little or no further net dimerisation was occurring. However, when starting with pure dimer, we did not detect any appreciable monomerisation (even in the presence of chaperones), arguing against an equilibrium reaction. Furthermore, the observation that RG-II dimerisation *can* proceed to completion with certain chaperones argues against the hypothesis that an equilibrium is reached at which dimerisation equals re-monomerisation. And we assume that the peptides are stable during the (up to) 24 h incubations. One possibility is that dimeric RG-II has a stronger affinity for some chaperones than does the monomer so that the remaining monomer cannot be adequately chaperoned for boron bridging to continue.

## Conclusions

Most of the pectic RG-II domains in living plants are covalently dimerised via borate diester bridges, essential for cell-wall assembly and correct porosity, but the formation of RG-II dimers from pure RG-II plus boric acid *in vitro* is extremely slow. Cationic chaperones greatly expedite boron bridging *in vitro*, probably by overcoming the mutual repulsion between neighbouring pairs of negatively charged RG-II molecules and/or by seating two RG-II molecules adjacent on the same chaperone molecule. Naturally relevant chaperones may include His-rich extensins [17], and highly effective artificial models include Pb^2+^ and polyhistidine [9,17,42].

We have now continued the search for naturally relevant chaperones, focusing on AGPs that are cationic overall and/or possess small, highly cationic domains. Cationic fragments of four such arabidopsis AGPs (AGP17, 18, 19 and 31) were effective, catalysing the boron bridging of RG-II *in vitro*. At least one of them (AGP31), was also effective as the native glycoprotein. We showed that all chaperones tested interact reversibly with RG-II, and so can be described as functioning catalytically — i.e. enzymically. To the best of our knowledge, such a role would be the first example of an enzyme activity (RG-II borate diesterase) acting on boron compounds.

The four unique AGPs studied here may serve a chaperoning role in the living plant cell, catalysing RG-II dimerisation within the Golgi cisternae and/or in the apoplast adjacent to the plasma membrane. In this way, RG-II and specific AGPs may be involved in guiding cell-wall assembly and hence cell expansion and development. Indeed, RG-II is present in the cell walls of all land-plants [40], and AGP-related sequences possessing cationic peptide domains also occur over a wide taxonomic range.

## Materials and methods

### Peptides and proteins

The peptides and artificial proteins used in this study (Table 1) were from Sigma–Aldrich (Poole, Dorset, U.K.) (polyhistidine; mean degree of polymerisation, DP, 106) and Neo Scientific (Cambridge, MA, U.S.A.) (hexahistidine; His_6_), both as chloride salts, and Thermo Fisher Scientific (Inchinnan, Renfrew, U.K.) (AGP17p, AGP18p, AGP19p1 and AGP19p2). AGP19p2 was designed to add flanking hydrophilic (Ala-Ser-Ala-Ser) regions at both ends of the highly cationic tridecapeptide stretch, mimicking the biological glycoprotein sequence, where the positively-charged tridecapeptide is flanked by hydrophilic, glycosylated -Ala-Hyp- repeats. Gum arabic was from Sigma–Aldrich (G9752), as was l-histidine (H-8125). For preparation of de-glycosylated gum arabic, 500 mg gum arabic was subjected to acid hydrolysis in 2 M trifluoroacetic acid (TFA) at 100°C for 1 h, then dialysed in 12 kDa-cutoff dialysis tubing against several changes of water until all TFA had been removed, and finally dried; the yield was 14 mg. The native AGP31 glycoprotein was isolated from etiolated hypocotyls as described by Hijazi et al. [41]. The AGP31p cationic peptide was synthesised by Millegen (Labège, France).

### RG-II isolation

Cultured *Rosa* cells [‘Paul's Scarlet’ rose, a complex hybrid; grown in boron-free medium [9] were rinsed in water, then alcohol-insoluble residue (AIR) was prepared by stirring in 75% ethanol at 20°C for 4–6 h twice. Pectin in the AIR was de-esterified with 1 M Na_2_CO_3_ at 4°C for 16 h, then the AIR suspension was slightly acidified with acetic acid, rinsed with water until neutral and freeze-dried. Endopolygalacturonase (10 U/ml; Megazyme, http://www.megazyme.com/) was added (∼50 µl/mg AIR) and incubated at 20°C for 16 h in 50 mM acetate (Na^+^, pH 4.8) [9]. The solubilised monomeric RG-II was purified by gel-permeation chromatography on Bio-Gel P-30 (Bio-Rad, Irvine, California, U.S.A.) in pyridine/acetic acid/water (1 : 1 : 98, containing 0.5% chlorobutanol), then dried *in vacuo*.

### Gel electrophoresis

PAGE was performed according to Chormova et al. [9]. To prepare one 26.4% polyacrylamide gel (83 × 73 × 0.75 mm), we mixed 834 µl water, 834 µl 1.5 M Tris (Cl^−^, pH 8.8), 3.33 ml 40% (w/v) acrylamide/bisacrylamide (29 : 1), 3.9 µl TEMED and 46.7 µl of freshly prepared 10% ammonium persulfate. The mixture was quickly poured, and a 10-tooth comb was inserted. The electrode buffer contained 50 mM Tris (free base) and 38 mM glycine, pH 9.2 (unadjusted). Samples (8–10 µl) were mixed with 2 µl loading buffer [0.63 M Tris–HCl containing 0.25% (w/v) bromophenol blue and 50% (v/v) glycerol, pH 8.8]. Electrophoresis was conducted at 200 V for 75 min. The gel was then fixed in ethanol/acetic acid/water (4 : 1 : 5) for 30 min, washed with water for 1 min three times, then treated successively with 400 µM sodium thiosulfate for exactly 1 min, water (3 × 1 min), freshly prepared 6 mM silver nitrate in 10 µM formaldehyde for 20 min, water (2 × 20 s) and 0.28 M Na_2_CO_3_ containing 8 µM sodium thiosulfate and 64 mM formaldehyde for 2–10 min. Colour development was stopped 30 s before the desired intensity had been reached by addition of 0.33 M Tris base in 2% v/v acetic acid for 10 min, and the gel was then immediately scanned. Colour development continued for ∼30–60 s in the stopping solution, the background turning dark yellow.

Where possible, we semi-quantified the silver-stained bands so that the % dimerisation could be reported. The method is described in detail in the legend to Supplementary Figure S2. In brief, the stained gel was scanned and the image converted to greyscale in Adobe Photoshop 5.5, then a standard rectangle was placed on each RG-II band, within which the mean luminosity was read. To correct for the background, we also measured the mean luminosity in a neighbouring blank area of the gel. Each RG-II band's mean luminosity was subtracted from that of the nearby blank zone, giving ‘Δ luminosity’ value, which was proportional to the mass of RG-II in the band (e.g. Supplementary Figure S3). A given mass of dimer gives stronger silver-staining than the same mass of monomer on the same gel, so separate standard curves were prepared for monomer and dimer. A ‘Δ luminosity’ value of 10 corresponded to 0.156 ± 0.014 µg of RG-II dimer or 0.314 ± 0.042 µg of monomer (mean ± SE of 14 gels). For each gel, a factor was obtained for converting Δ luminosity to mass for monomer bands by reference to markers and/or any reaction mixtures in which essentially all the RG-II was still monomeric; and for dimers it was obtained by reference to markers and/or any reaction mixture in which almost all the RG-II had been dimerised. When no such dimer reference band was available on a particular gel, a comparably stained gel was used. It should be noted that the background staining of these gels is slightly uneven, so some faint bands ended up with an apparent negative mass, resulting in the ‘% dimerisation’ being scored as slightly above 100% or below 0%, [e.g. in Figure 3, Figure 8e and Supplementary Figure S5b].

### *In-vitro* dimerisation of RG-II

In studies of dimerisation *in vitro*, the RG-II monomer (16 or 20 µM) was incubated at 20°C for 16 h in an excess (1.2 mM) of H_3_BO_3_, with or without 0.5 mM PbNO_3_, and with or without various peptides (Table 1) or native AGP31 glycoprotein (concentrations indicated on Figs.). All reaction mixtures were buffered at pH 4.8 with 50 mM acetate (Na^+^); the optimal pH for dimerisation in the presence of peptides is 2.5–4.8 (Begum and Fry, in preparation). Samples were analysed by PAGE without further preparation; neither the presence of chaperones nor the buffer affected electrophoresis mobility.

### Prediction of peptide and protein isoelectric points

The pI values of amino acid sequences were estimated (Supplementary Fig. S1, Table 1) by online services. Unless otherwise stated, we used IPC 2.0 (www.ipc2-isoelectric-point.org), which makes use of a combination of deep learning and support vector regression models [47]. Other websites [‘Compute pI/Mw tool’ (https://web.expasy.org/compute_pi/), ‘Bachem Peptide Calculator’ (https://www.bachem.com/knowledge-center/peptide-calculator/), Prot pi (with ‘ProMoST’ data source for *p*K_a_ values; https://www.protpi.ch/Calculator/ProteinTool), and Prot pi (with ‘native’ data source for *p*K_a_ values)] gave rather different predictions (Supplementary Table S1).

## Data Availability

All data relevant to this manuscript are provided within the paper as tables, figures and supplementary file. We have not included any structural/crystallographic data (for either macromolecular structures or small molecules), protein and nucleic acid sequence data other than what are already published on universally accessible websites, functional genomics and molecular interactions/proteomics/metabolomics data, computational models, or genetics data (genetic polymorphisms; genotype data).

## Open Access

Open access for this article was enabled by the participation of University of Edinburgh in an all-inclusive *Read & Publish* agreement with Portland Press and the Biochemical Society under a transformative agreement with JISC.

## Abbreviations

AGP: arabinogalactan-protein
AGP17p etc.: peptides of AGPs
AIR: alcohol-insoluble residue
DP: degree of polymerisation; GPI, glycosylphosphatidylinositol
PAGE: polyacrylamide gel electrophoresis
RG-II: rhamnogalacturonan-II
