# Supplementary material for: An Arabidopsis thaliana arabinogalactan-protein (AGP31) and several cationic AGP fragments catalyse the boron bridging of rhamnogalacturonan-II
Source: Biochem J. 2022 Sep 23;479(18):1967–84. doi: 10.1042/BCJ20220340 (PMC9555800; doi:10.1042/BCJ20220340)
Supplement: Supplementary Material [file BCJ-479-1967-s1.pdf]

An *Arabidopsis thaliana* arabinogalactan-protein (AGP31) and several cationic AGP fragments catalyse the boron bridging of rhamnogalacturonan-II

Dayan Sanhueza, Rifat Ara Begum, Cécile Albenne, Elisabeth Jamet, and Stephen C. Fry

Supplementary file

Contents

Figure S1. Sequences of selected arabidopsis arabinogalactan-proteins

Figure S2. Method for approximately quantifying silver-stained bands in Photoshop

Figure S3. Quantification of silver-stained RG-II bands after polyacrylamide gel electrophoresis

Figure S4. Effect of boron concentration on the polyhistidine-enhanced dimerisation of RG-II

Figure S5. Comparison of various cationic chaperones for catalysis of RG-II dimerisation

Figure S6. Deglycosylated and intact gum arabic as potential RG-II chaperones

Table S1. Variation in isoelectric point prediction between different online services

AGP17 pI 5.7, 13.55 kDa

MTRNILLTVTLICIVFITVGG

QSPATAPIHSPSTSPHKPKPTSPAISPAAPTPESTEAPAKTPVEAPVEAPPSPTPASTPQISPPAPSP
EADTPSAPEIAPSADVPAPALT**KHKKKTKKHK**TAPAPGPASELLSPPAPPGEAPGPGPSDAFSPAADD
QS—**GPI**

GAQRISVVIQMVGAAAIAWSLLVLAF

AGP18 pI 7.6, 15.69 kDa

MDRNFLLTVTLICIVVAGVGG

QSPISSPTKSPTTPSAPTTSPTKSPAVTSPTTAPAKTPTASASSPVESPKSPAPVSESSPPPTPVPES
SPPVPAPMVSSPVSSPPVPAPVADSPPAPVAAPVADVPAPAPS**KHKKTTKKSKKH**QAAPAPAPELLGP
PAPPTESPGPNSDAFSPGPSADDQSG—**GPI**

AASTRVLRNVAVGAVATAWAVLVMAF

AGP19 pI 9.2, 16.58 kDa

MESNSIIWSLLLASALISSFSVNA

QGPAASPVTSTTTAPPPTTAAPPTTAAPPPTTTTPPVSAAQPPASPVTPPPAVTPTSPPAPKVAPVIS
PATPPPQPPQSPPASAPTVSPPPVSPPPAPTSPPPTPASPPPAPASPPPAPASPPPAPVSPPPVQAPS
PISLPPAPAPAPT**KHKRKHKHKRHHH**APAPAPIPPS—**GPI**

PPSPPVLTDPQDTAPAPSPNTNGGNALNQLKGRAVMWLNTGLVILFLLAMTA

AGP31 pI 9.1, 35.91 kDa

MGFIGKSVLVSLVALWCFTSSVFT

EEVNHKTQTPSLAPAPAP**YHHGHHHPHPPHHHHPHPHPHPHP**PAKSPVKPPVKAPVSPPAKPPVKPPV
YPPTKAPVKPPTKPPVKPPVSPPAKPPVKPPVYPPTKAPVKPPTKPPVKPPVYPPTKAPVKPPTKPPV
KPPVYPPTKAPVKPPTKPPVKPPVSPPAKPPVKPPVYPPTKAPVKPPVSPPTKPPVTPPVYPPKF**N**RS
LVAVRGTVYCKSCKYAAFNTLLGAKPIEGATVKLVCKSKK**N**ITAETTTDKNGYFLLLAPKTVTNFGFR
GCRVYLVKSKDYKCSKVSKLFGGDVGAELKPEKKLGKSTVVVNKLVYGLFNVGPFAFNPSCPK

Figure S1. Sequences of selected arabidopsis arabinogalactan-proteins.
Mature proteins after cleavage of proposed signal peptide and insertion of proposed GPI (glycosyl-phosphatidylinositol) anchor. Isoelectric point predictions (by IPC 2.0) and molecular mass are after removal of the N-terminal signal peptide, and without glycosylation. **Underlined, bold** stretches are the peptides tested in the present work. *Italic* stretches are proposed to be removed in the mature proteins. **N** = putative Asn *N*-glycosylation site. Colour code:

P	single proline	S,T	serine & threonine	H	histidine
PP	double proline	D	aspartic acid	K	lysine
PPP	triple proline	E	glutamic acid	R	arginine

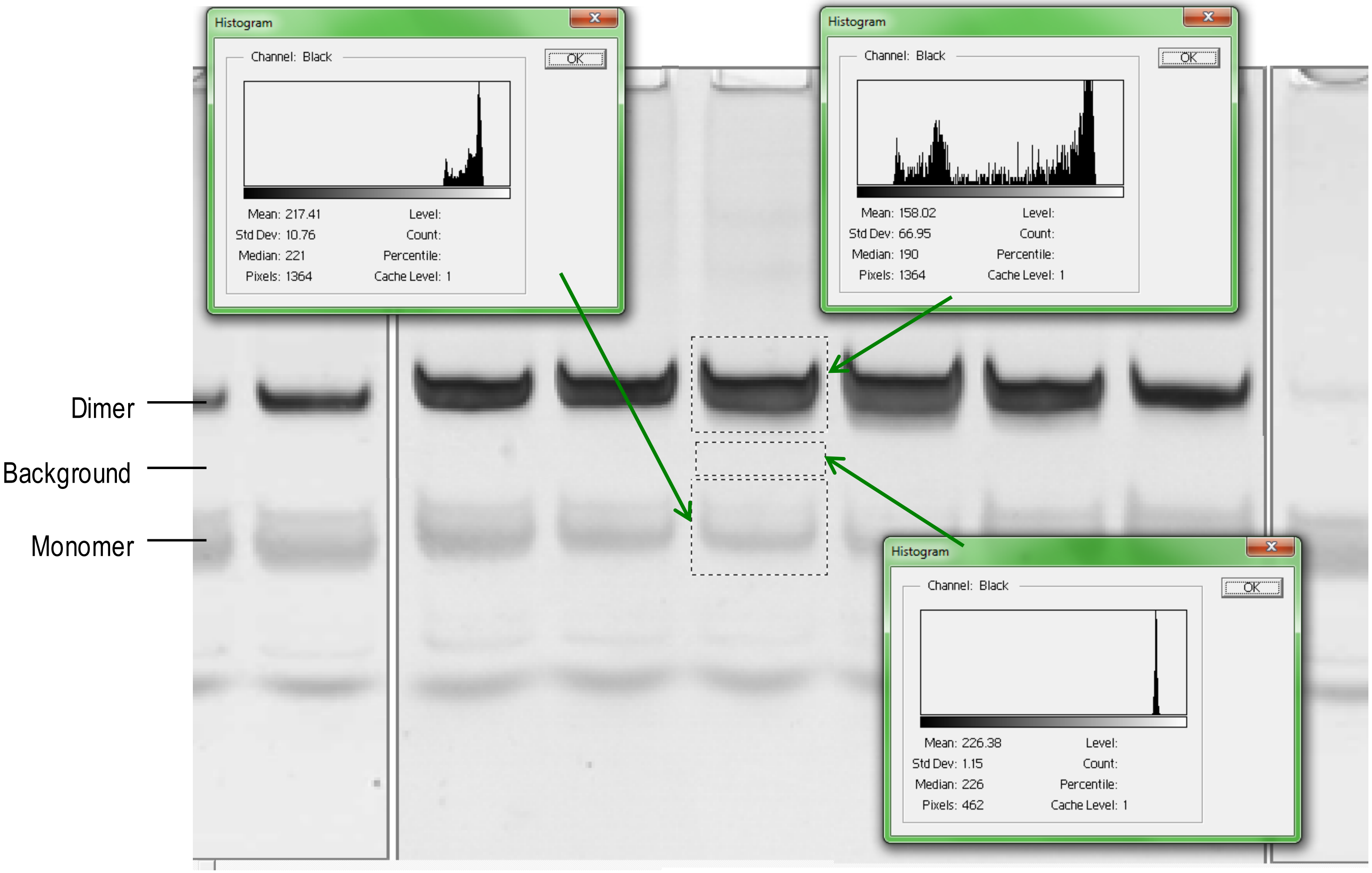

Figure S2. Method for approximately quantifying silver-stained bands in Photoshop.

As an example, this diagram shows part of Fig. 7a, indicating how the bands in the 50 µg/ml AGP19p1 lane were semi-quantified. In Adobe Photoshop 5.5, the scan was converted to greyscale, then a standard rectangle was placed on each RG-II band, and the histogram facility was applied, giving a mean luminosity (which is expressed by the software per unit area; in this case, 217.41 and 158.02 for the monomer and dimer, respectively). The rectangle always ha d the same area for every RG-II band on a given gel, and was always large enough to accommodate the whole band. Each reading thus gave the mean luminosity of the RG-II band plus an area of background gel. To correct for the background, we also placed a rectangle on the mid-point between each pair of RG-II bands and recorded this mean luminosity reading (226.38 in this case). In some cases, the background rectangle was smaller than the rectangle used for the RG-II bands, as illustrated here, so that no RG-II tail was included. The mean luminosity is not affected by this difference in area. The mean luminosity value returned for pure white and black zones of a n image are 255.0 and 0.0 respectively. Each RG-II band's mean luminosity was subtracted from its nearest background, and the result (Δ luminosity) was used as a measure of the mass of RG-II in the band. For each gel, a separate conversion factor was determined. As shown in Fig. S3, mass was approximately proportional to the Δ luminosity value. A given mass of dimer gives a much higher value than the same mass of monomer on the same gel, so separate conversion factors were used for monomers and dimers on each gel.

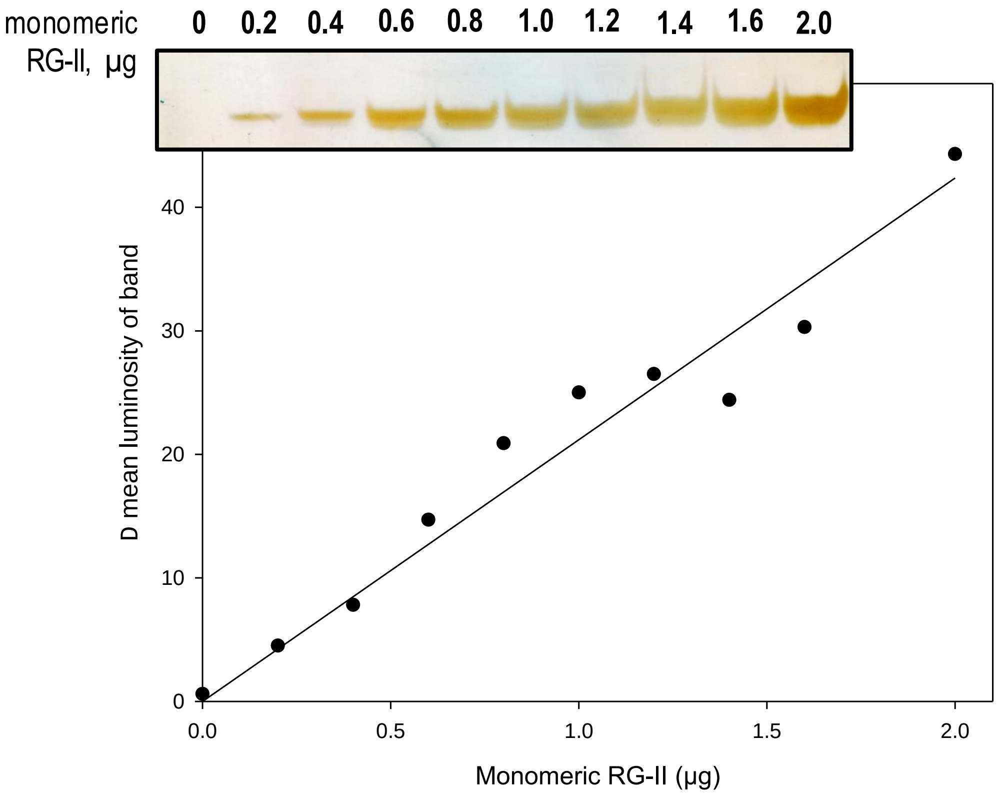

Figure S3. Quantification of silver-stained RG-II bands after polyacrylamide gel electrophoresis.

Various loadings of monomeric rhamnogalacturonan-II, purified from arabidopsis cell-suspension cultures [9], were analysed by PAGE and silver-stained. In Photoshop, the image was converted to greyscale and the mean luminosity within a standard rectangle was measured and corrected for the nearest-neighbour background area of the gel (see Fig. S2).

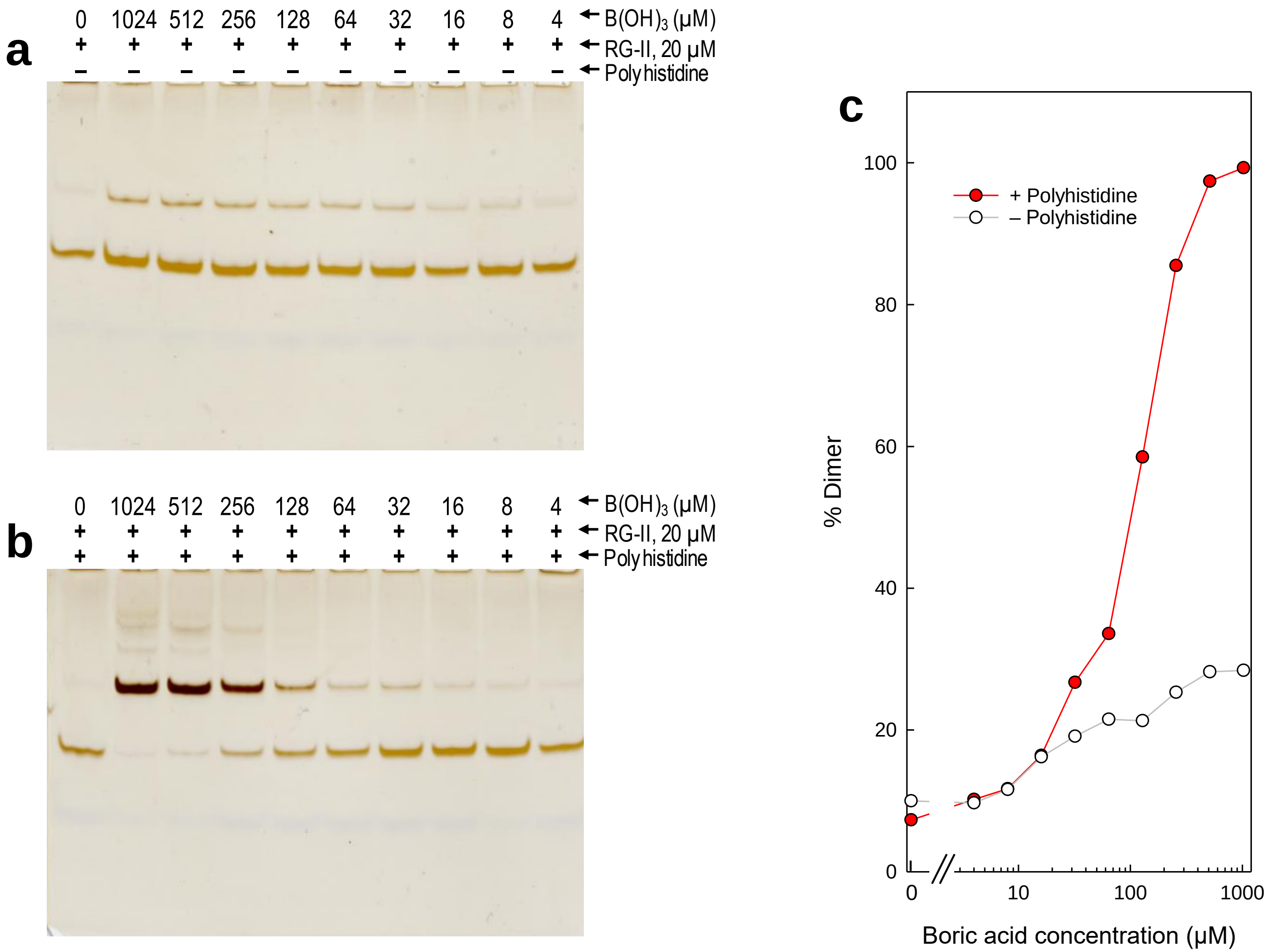

Figure S4. Effect of boron concentration on the polyhistidine-enhanced dimerisation of RG-II.

Reaction mixtures contained 50 µg/ml monomeric RG-II (20 µM) with 0–1024 µM boric acid and 50 mM acetate (Na^+, pH 4.8), without (a) or with (b) polyhistidine.Cl^- (50 µg/ml; 2.8 µM). After 4 h at 20°C, 0.8 µg of the RG-II was analysed by PAGE followed by silver staining. (c) Quantification of the extent of dimerisation.

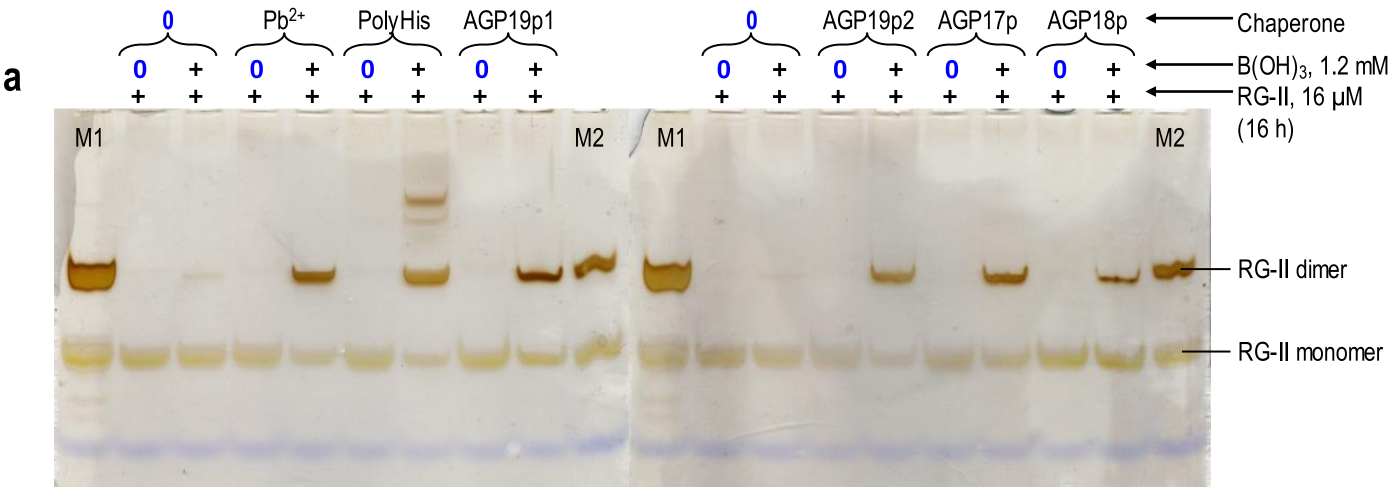

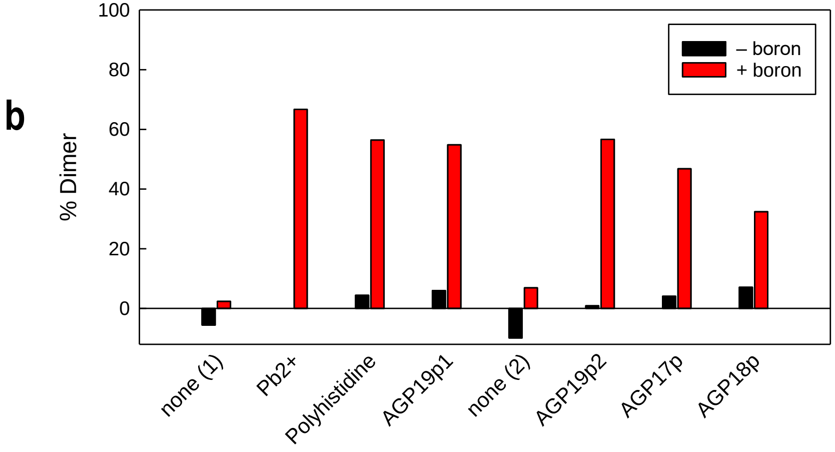

Figure S5. Comparison of various cationic chaperones for catalysis of RG-II dimerisation.

Reaction mixtures contained 80 µg/ml monomeric RG-II with 0 or 1.2 mM boric acid and 50 mM acetate (Na^+, pH 4.8) plus a chaperone: 0.5 mM Pb^{2+} or 50 µg/ml* polyhistidine, AGP17p, AGP18p, AGP19p1 or AGP19p2. Controls lacked any chaperone. After 16 h at 20 °C, 0.8 µg of the RG-II was analysed by PAGE followed by silver staining. (a) Stained gel, (b) quantification of the % dimerisation. Markers were: M1, 1.2 µg monomeric RG-II + 1.2 µg dimeric RG-II; M2, 0.8 µg monomeric RG-II + 0.8 µg dimeric RG-II.

*The 50 µg/ml peptide solutions represent 2.7 µM poly-His, 39 µM AGP17p, 34 µM AGP18p, 28 µM AGP19p1 and 20 µM AGP19p2.

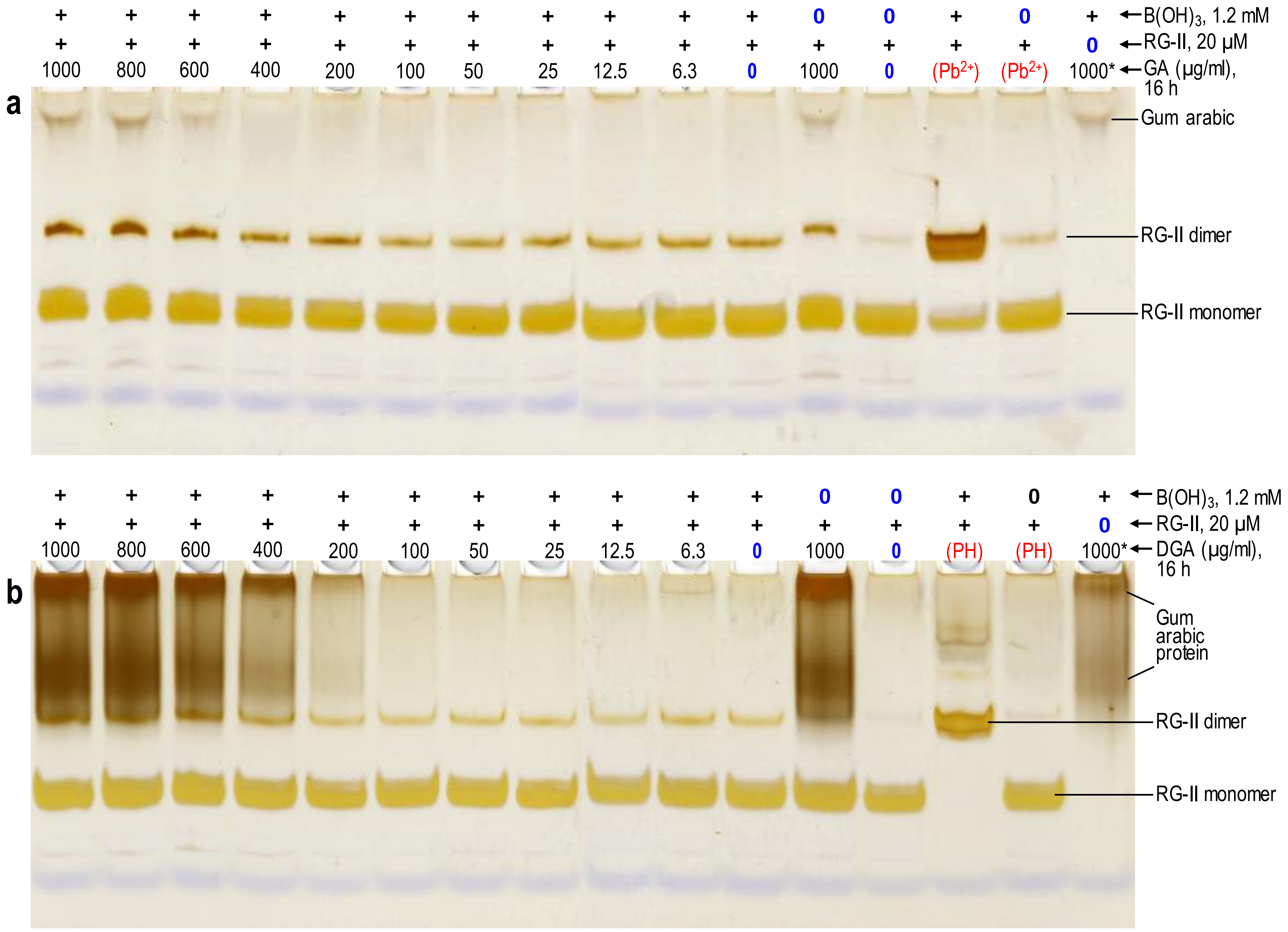

Figure S6. Deglycosylated and intact gum arabic as potential RG-II chaperones.

Reaction mixtures contained 100 µg/ml RG-II monomer (≈20 µM), 1.2 mM boric acid, 0–1000 µg/ml of **(a)** gum arabic (GA) or **(b)** the protein core of deglycosylated gum arabic (GAPr), and 50 mM acetate (Na^+) buffer, pH 4.8. Controls lacked boric acid or RG-II, or contained 0.5 mM Pb^{2+} or 50 µg/ml polyhistidine (PH) as alternative chaperones. After 16 h at 20 °C, 0.8 µg of the RG-II was analysed by PAGE followed by silver staining. The right-hand lanes (marked 1000*) contained 1000 µg/ml GA or GA Pr plus 50 µg/ml polyhistidine. The bands in (a) are quantified in Fig. 3a.

Table S1. Variation in isoelectric point prediction between different online services*

Name	Sequence[1]	pI (Expasy)[2]	pI (Bachem)[3]	pI (ProtPi) (ProMoST)[4]	pI (ProtPi) (Native)[5]	pI (IPC 2.0)[6]
Histidine	**H**	6.74	7.90	6.59	6.59	7.24
His_6	**HHHHHH**	7.21	8.19	7.26	7.48	7.34
Poly-histidine	**HHHHHHHHHHHHHHHHHHHHHHHHHHHHHHHHHHHHHHHHHHHHHHHHHHHH<br>HHHHHHHHHHHHHHHHHHHHHHHHHHHHHHHHHHHHHHHHHHHHHHHHHHHH<br>HH**	8.10	8.94	8.16	8.64	7.77
AGP17p	**KHKKKTKKHK**	10.78	11.52	10.71	11.20	8.18
AGP18p	**KHKKTTKKSKKH**	10.78	11.52	10.61	11.23	8.21
AGP19p1	**KHKRKHKHKRHHH**	12.04	12.56	12.51	12.53	8.84
AGP19p2	**ASASKHKRKHKHKRHHHASAS**	12.04	12.56	12.51	12.54	8.84
AGP31p	**YHHGHHHPHPPHHHHPHPHPHPHP**	7.44	8.32	7.32	7.75	7.96
AGP31	**EEVNHKTQTPSLAPAPAPYHHGHHHPHPPHHHHPHPHPHPHPPAKSPVKPPV<br>KAPVSPPAKPPVKPPVYPPTKAPVKPPTKPPVKPPVSPPAKPPVKPPVYPPT<br>KAPVKPPTKPPVKPPVYPPTKAPVKPPTKPPVKPPVYPPTKAPVKPPTKPPV<br>KPPVSPPAKPPVKPPVYPPTKAPVKPPVSPPTKPPVTPPVYPPKFNRSLVAV<br>RGTVYCKSCKYAAFNTLLGAKPIEGATVKLVCKSKKNITAETTTDKNGYFLL<br>LAPKTVTNFGFRGCRVYLVKSKDYKCSKVSKLFGGDVGAELKPEKKLGKSTV<br>VVNKLVYGLFNVGPFAFNPSCPK**	10.19	10.66	10.00	10.64	9.14

*Calculated after *in-silico* removal of the putative *N*-terminal signal sequence but without proline hydroxylation and glycosylation.
[1] Amino acid residues with cationic and anionic side-chains are highlighted in shades of blue and red respectively.
[2] Isoelectric point estimated by 'Compute pI/Mw tool' (https://web.expasy.org/compute_pi/).
[3] Isoelectric point estimated by 'Bachem Peptide Calculator' (https://www.bachem.com/knowledge-center/peptide-calculator/).
[4] Isoelectric point estimated by Prot pi (with 'ProMoST' data source for pK_a values) (https://www.bachem.com/knowledge-center/peptide-calculator/).
[5] Isoelectric point estimated by Prot pi (with 'native' data source for pK_a values) (https://www.bachem.com/knowledge-center/peptide-calculator/).
[6] Isoelectric point estimated by IPC 2.0 (peptide if DP<60, protein if DP>60) (www.ipc2-isoelectric-point.org).
